# In Vitro Rumen Simulations Show a Reduced Disappearance of Deoxynivalenol, Nivalenol and Enniatin B at Conditions of Rumen Acidosis and Lower Microbial Activity

**DOI:** 10.3390/toxins12020101

**Published:** 2020-02-05

**Authors:** Sandra Debevere, An Cools, Siegrid De Baere, Geert Haesaert, Michael Rychlik, Siska Croubels, Veerle Fievez

**Affiliations:** 1 Department of Pharmacology, Toxicology and Biochemistry, Faculty of Veterinary Medicine, Ghent University, Salisburylaan 133, 9820 Merelbeke, Belgium; sandra.debevere@ugent.be (S.D.); siegrid.debaere@ugent.be (S.D.B.); siska.croubels@ugent.be (S.C.); 2 Department of Animal Sciences and Aquatic Ecology, Faculty of Bioscience Engineering, Ghent University, Coupure links 653, 9000 Ghent, Belgium; 3 Department of Nutrition, Genetics and Ethology, Faculty of Veterinary Medicine, Ghent University, Heidestraat 19, 9820 Merelbeke, Belgium; an.cools@ugent.be; 4 Department of Plants and Crops, Faculty of Bioscience Engineering, Ghent University, Valentin Vaerwyckweg 1, 9000 Ghent, Belgium; geert.haesaert@ugent.be; 5 Chair of Analytical Food Chemistry, Technical University of Munich, Maximus-von-Imhof-Forum 2, 85354 Freising, Germany; michael.rychlik@tum.de

**Keywords:** mycotoxins, maize silage, SARA, lactation stage, rumen fluid, UPLC-MS/MS

## Abstract

Ruminants are generally considered to be less susceptible to the effects of mycotoxins than monogastric animals as the rumen microbiota are capable of detoxifying some of these toxins. Despite this potential degradation, mycotoxin-associated subclinical health problems are seen in dairy cows. In this research, the disappearance of several mycotoxins was determined in an in vitro rumen model and the effect of realistic concentrations of those mycotoxins on fermentation was assessed by volatile fatty acid production. In addition, two hypotheses were tested: (1) a lower rumen pH leads to a decreased degradation of mycotoxins and (2) rumen fluid of lactating cows degrade mycotoxins better than rumen fluid of non-lactating cows. Maize silage was spiked with a mixture of deoxynivalenol (DON), nivalenol (NIV), enniatin B (ENN B), mycophenolic acid (MPA), roquefortine C (ROQ-C) and zearalenone (ZEN). Fresh rumen fluid of two lactating cows (L) and two non-lactating cows (N) was added to a buffer of normal pH (6.8) and low pH (5.8), leading to four combinations (L6.8, L5.8, N6.8, N5.8), which were added to the spiked maize substrate. In this study, mycotoxins had no effect on volatile fatty acid production. However, not all mycotoxins fully disappeared during incubation. ENN B and ROQ-C disappeared only partially, whereas MPA showed almost no disappearance. The disappearance of DON, NIV, and ENN B was hampered when pH was low, especially when the inoculum of non-lactating cows was used. For ZEN, a limited transformation of ZEN to α-ZEL and β-ZEL was observed, but only at pH 6.8. In conclusion, based on the type of mycotoxin and the ruminal conditions, mycotoxins can stay intact in the rumen.

## 1. Introduction

Mycotoxins are secondary fungal metabolites that are harmful to animals and humans [1]. Monogastric animals are considered to be more susceptible to the toxic effects of mycotoxins than ruminants as the rumen microbiota are capable to degrade some of those toxic molecules to less toxic metabolites [2,3,4]. In addition, intrinsic rumen fluid factors, such as bacterial and yeast cell walls and feed particles, can deactivate mycotoxins (e.g., aflatoxins and zearalenone) by binding [5,6,7,8,9,10,11]. However, in high-productive dairy cows, mycotoxin-associated subclinical health problems may occur, reflected by vague and non-specific symptoms and periodic decrease in milk production [2]. One possible reason could be the higher proportion of maize silage in the ration of higher yielders [2], which is more vulnerable to contamination with multiple mycotoxins than e.g., grassland products [12,13,14]. However, in Belgium and the Netherlands, maize silage is also given to non-lactating cows instead of grassland products as maize silage is low in calcium and potassium which diminish the incidence of milk fever at the beginning of lactation [15,16]. In addition, the highly fermentable ration of lactating cows in combination with a higher feed intake leads to a higher rumen passage rate [17] and a higher incidence of metabolic disorders such as subacute ruminal acidosis (SARA), associated with a microbial shift in the rumen [18]. These factors can lead to an impaired detoxification of mycotoxins by the rumen microbiota so that mycotoxins can reach the postruminal parts of the gastrointestinal tract, may impair gut health and the gut barrier function and/or can be absorbed, entering systemic circulation and exerting their toxic effects on the animal.

In the period from 2016 till 2018, maize samples from 257 maize fields across Flanders (Belgium), were analyzed on mycotoxin contamination before ensiling. The mycotoxins most often found in these samples were nivalenol (NIV), deoxynivalenol (DON), zearalenone (ZEN), and enniatin B (ENN B) and were found in respectively 99.2%, 85.6%, 49.8%, and 36.2% of all samples [19]. Of those samples, 2.3% and 7.8% exceeded the European Union reference values for complementary and complete feedstuffs for calves (<4 months) for DON and ZEN, respectively [20]. In 21 maize silage samples from Belgium, DON (in all samples), ZEN (in 90% of the maize silage samples), and ENN B (in 86% of silage samples) were also frequently found. In addition, roquefortine-C (ROQ-C, in all moldy hot spots and in 62% of non-moldy parts) and mycophenolic acid (MPA, 95% in non-moldy parts and in 86% of moldy hot spots) were highly prevalent [21,22,23]. It is reported that, on the one hand, in healthy ruminants DON and NIV are converted almost completely to their less toxic metabolites deepoxy-DON (DOM-1) and deepoxy-NIV by the rumen microbiota, with conversion rates up to 62–99% for DON [24,25,26] and up to 78–82% for NIV [25]. On the other hand, studies have also shown that ZEN can be converted by the rumen microbiota for 25% to 90% into the more toxic metabolite α-zearalenol (α-ZEL) and the less toxic metabolite β-zearalenol (β-ZEL) [3,24,27,28,29]. Mycophenolic acid and ROQ-C are reported to stay mainly intact in an in vitro rumen simulation study after 48 h of incubation, so these mycotoxins could reach the intestinal tract intact after ingestion [30]. To the authors’ knowledge, research about the detoxification of ENN B in the rumen is still lacking.

Until now, studies investigating the impact of low ruminal pH or origin of rumen inoculum (e.g., rumen fluid of non-lactating versus lactating cows) on the detoxification capacity for mycotoxins are scarce. Some research with DON points already towards the importance of pH and microbial activity in the degradation capacity of microbiota. A study of He et al. demonstrated that contents of the large intestine of chickens could not detoxify DON anymore in vitro when the buffer pH was below 5.2 [31]. In addition, Valgaeren et al. demonstrated a close relationship between the detoxification of DON and the functional ruminal microbiota [14]. As the microbial activity of rumen fluid of non-lactating cows assumed to be lower than the microbial activity of inoculum from lactating cows, it is presumed that mycotoxin detoxification differs between both inocula.

In this research, the disappearance of several mycotoxins was determined in an in vitro rumen model and the effect of realistic concentrations of those mycotoxins on fermentation was assessed by volatile fatty acid (VFA) production. In addition, two hypotheses were tested: (1) a lower rumen pH leads to a decreased degradation of mycotoxins and (2) microbiota in rumen fluid of lactating cows degrade mycotoxins better than rumen fluid of non-lactating cows. The mycotoxins DON, NIV, ZEN, MPA, ROQ-C and ENN B were investigated. Moreover, DOM-1 as a metabolite of DON and α-ZEL, β-ZEL, zearalanone (ZAN), α-zearalanol (α-ZAL) and β-zearalanol (β-ZAL) as metabolites of ZEN were monitored as these can be formed in the rumen [3,27,28,29,32,33] and analytical standards are commercially available.

## 2. Results and Discussion

### 2.1. Incubation Set-Up

For this in vitro rumen incubation, the rumen fluid/buffer ratio was set at 1/4 to obtain a stable pH as well as in vitro gas and fermentation profiles that are comparable with the in vivo rumen fermentation. This ratio was also frequently used in other in vitro rumen studies. Pell and Schofield (1993) mentioned that 20% of inoculum is sufficient to ensure the maximum rate of fiber digestion [34]. Hence, relative effects, i.e., changes of mycotoxin disappearance between incubations with different pH or inoculum, could be assessed through this simulation system.

In the current in vitro study, a kinetic profile of mycotoxin disappearance was established within a 48 h time frame. This is considerably longer than the in vivo retention time of digesta in the rumen, but in line with other in vitro experiments studying mycotoxin degradation in vitro [25,31,35,36]. It should be noted that direct extrapolation of in vitro incubation times to in vivo rumen retention times should not be made. The batch incubation set-up is a static model with some lag time until the microbiota are fully active in the incubation flasks. This lag time might be particularly important regarding the microbial breakdown of complex molecules such as mycotoxines when the inoculum is obtained from animals which are not exposed to mycotoxin contaminated feed.

### 2.2. Volatile Fatty Acids

The final model for total net VFA production covers six fixed effects model parameters with replicate as random intercept. An overview of the parameter estimates can be found in Table 1.

**Figure 1:**
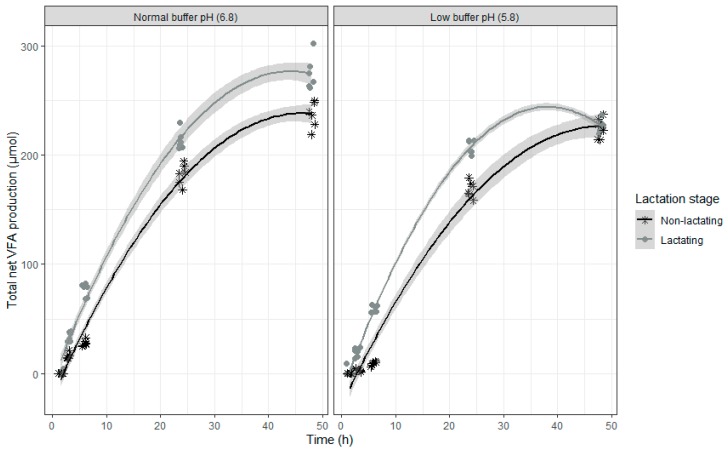
Total net volatile fatty acid (VFA) production (µmol) during an in vitro rumen simulation study. In this study, the effect of rumen fluid pH (5.8 or 6.8), lactation stage (inoculum of non-lactating or lactating cows) and presence/absence of mycotoxins on the total VFA production was investigated during an incubation period of 48 h. In this figure, no distinction was made between samples with or without mycotoxins as this had no effect on total net VFA production. The grey bands correspond to the 95% confidence level interval for predictions.

During the incubation, a fast increase of VFA concentrations could be detected (Figure 1 and Table 1). However, the VFA production slowed down during the last incubation hours. The inoculum of lactating cows led to a higher total net VFA production compared to the inoculum of non-lactating cows. In contrast to this, the rumen fluid-buffer mixture with a low pH (5.8) showed a lower total net VFA production compared to the rumen fluid-buffer mixture with a normal pH (6.8). The presence or absence of the mycotoxin mixture had no influence on the total net VFA production.

The higher VFA production of the inoculum of lactating cows compared to the inoculum of non-lactating cows confirms the assumption of an enhanced microbial activity of inoculum of lactating cows. A lower production of VFA at buffer pH below 6.0 was expected as such circumstances might inhibit pH-sensitive ruminal bacteria (e.g., cellulolytic bacteria) [37,38,39].

A negative effect of mycotoxins on VFA production has already been observed in several experiments. However, these studies generally used higher mycotoxin concentrations that are rarely observed in ruminant feeds. In a study of Gallo et al., ROQ-C and MPA were incubated at a concentration range of 0.01–2.00 µg of each mycotoxin per mL diluted rumen fluid and provoked a quadratic decrease of VFA production as the concentration of the mycotoxins increased [30]. However, the highest concentration in the latter study was substantially higher than the ROQ-C and MPA concentration in our study (ROQ-C: 30 ng/mL; MPA: 90 ng/mL). Jeong et al. also showed a lower total VFA production in an in vitro batch incubation study when incubated with maize starch contaminated with 40 mg DON/kg [32], which is approximately three times the European maximum guidance value in maize by-products [20]. However, when 1 µg/mL NIV and 2 µg/mL DON were incubated in a rumen simulation technique (RUSITEC) model, no effect was seen on VFA production, despite the considerably higher concentrations compared with our study, containing only 600 ng NIV and 120 ng DON per mL rumen fluid-buffer mixture [40]. Our findings are also in accordance with an in vivo study performed by Dänicke et al. whereby cows were fed DON- and ZEN-contaminated feed at concentrations of 8.05 mg DON per kg wheat and 0.26 mg ZEN per kg wheat with wheat representing half of the daily diet on dry matter (DM) basis [28]. In this study, no change in VFA concentrations were observed. Hence, a negative effect of the tested mycotoxins at a realistic contamination level on VFA production in the rumen is not expected.

As VFA production increases, pH of the samples may decrease if the buffering capacity is exceeded. In this study, the buffer showed a good buffering capacity as the mean pH of the samples with high buffer pH remained at 6.5 (±0.08) and the mean pH of the samples with low buffer pH remained at 5.7 (±0.05) at the end of the incubation.

### 2.3. Influence of Lactation Stage and pH on Disappearance of Mycotoxins

An overview of all mycotoxin concentrations determined per incubation time point and per treatment can be found in Table S1. In the following paragraphs, mycotoxin concentrations are expressed relative to the maximal (free) mycotoxin concentration detected during the total duration (48 h) of the in vitro rumen simulation study. This approach facilitates presentation and interpretation of the results as mycotoxins might adsorb to feed or other particles in rumen fluid. Indeed, bacteria and yeast cell walls could be involved in adsorbing aflatoxins, ZEN, and fumonisins [5,6,7,8,9,10,11,41], while, in our former study, ENN B, ROQ-C, and ZEN adsorbed to maize silage and MPA to rumen fluid particles [42]. Adsorbed mycotoxins are possibly released during the in vitro incubation, e.g., when feed particles are degraded. As such, mycotoxin concentrations could increase during the incubation as only the free mycotoxin fraction was determined.

#### 2.3.1. Mycotoxins Completely Disappearing at Normal pH: DON and NIV

The final models for relative DON or NIV concentration compared to the maximal DON or NIV concentration both count nine fixed effects model parameters with replicate as random intercept and time as random slope. An overview of the parameter estimates for each model can be found in Table 2.

**Figure 2:**
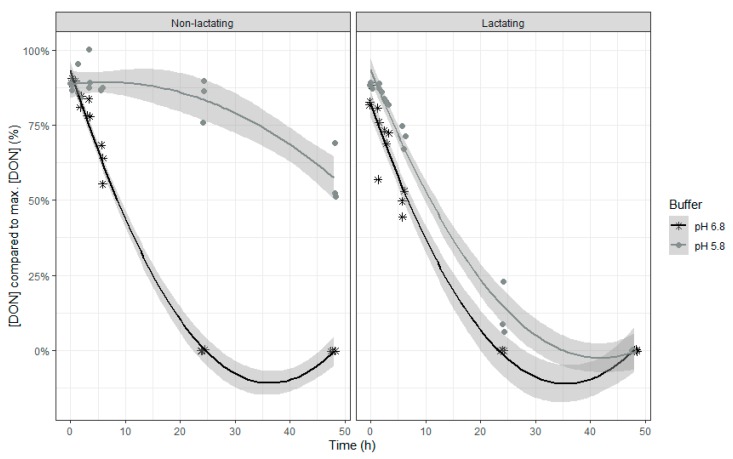
Deoxynivalenol concentration expressed relative to the maximum DON concentration during an in vitro rumen simulation study. In this study, the effect of rumen fluid pH (5.8 or 6.8) and lactation stage (inoculum of non-lactating or lactating cows) on the disappearance of DON was investigated during an incubation period of 48 h. The grey bands correspond to the 95% confidence level interval for predictions.

**Figure 3:**
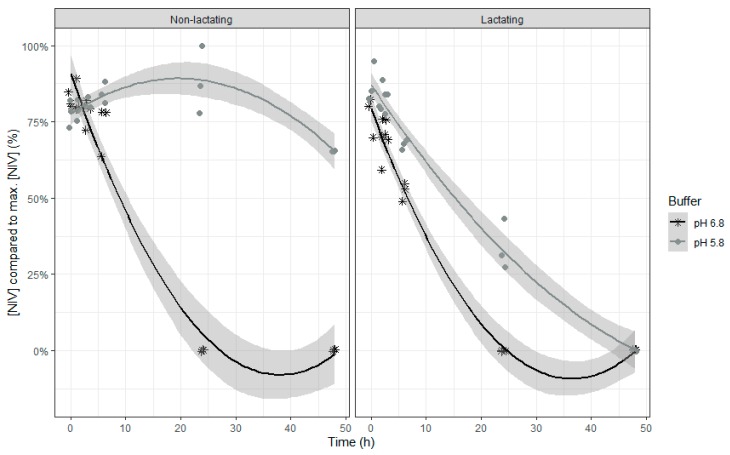
Nivalenol concentration expressed relative to the maximum NIV concentration during an in vitro rumen simulation study. In this study, the effect of rumen fluid pH (5.8 or 6.8) and lactation stage (inoculum of non-lactating or lactating cows) on the disappearance of NIV was investigated during an incubation period of 48 h. The grey bands correspond to the 95% confidence level interval for predictions.

Both models show similar characteristics, which is not surprising as DON and NIV have almost the same chemical structure. When inoculum of lactating cows was used, DON and NIV concentrations generally were lower. For DON, the lower buffer pH led also to an overall higher concentration of this mycotoxin. The relative DON and NIV concentrations decrease over time, with a slower disappearance at the end of the incubation (Table 2 and Figure 2 (DON) and Figure 3 (NIV)). It should be noted that negative values produced by the model are obviously irrelevant and correspond with 0%. A complete disappearance of both mycotoxins at normal pH was seen after 24 h of incubation. However, when the pH of the buffer was low, the DON and NIV disappearance was slower. Low pH particularly reduced DON and NIV disappearance when rumen fluid of non-lactating cows was used, while the negative effect was more moderate when the inoculum of lactating cows was used.

The final model for relative DOM-1 molar concentration compared to the maximal DON molar concentration counts, just like its parent compound DON, nine fixed effects model parameters with replicate as random intercept and time as random slope. An overview of the parameter estimates can be found in Table 3.

**Figure 4:**
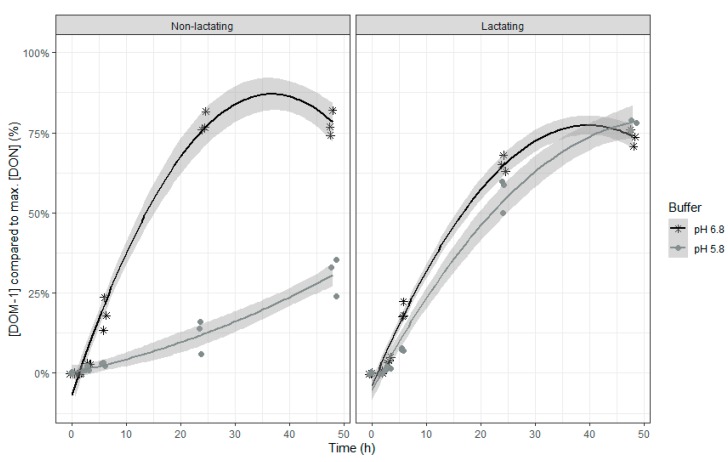
Molar DOM-1 concentration expressed relative to the maximum molar DON concentration during an in vitro rumen simulation study. In this study, the effect of rumen fluid pH (5.8 or 6.8) and lactation stage (inoculum of non-lactating or lactating cows) on the formation of the metabolite DOM-1 out of the parent molecule DON was investigated during an incubation period of 48 h. The grey bands correspond to the 95% confidence level interval for predictions.

The relative DOM-1 concentration increased over time, with a slower formation at the end of the incubation (Table 3 and Figure 4). However, when the pH of the buffer was low, the DOM-1 formation was much slower, except when using rumen fluid of lactating cows.

The DOM-1 accumulation relative to the incubation time corresponds to the inverse of the disappearance over time of its parent compound DON for all four combinations. Except for the combination of low buffer pH and inoculum of non-lactating cows, after 48 h of incubation, DON had completely disappeared with 75% recovered as free DOM-1. After 48 h incubation at the low pH and with inoculum of non-lactating cows, DON disappearance and DOM-1 accumulation represented 40% and 30% of the maximum DON concentration, respectively. This means that in all situations about 25% of the disappeared DON is not recovered as free DOM-1. Although a full recovery was not reached in this study, the recovery of DON + DOM-1 of 75–90% is in line with other in vitro rumen studies. In a study of King et al. (1984), a total recovery of DON + DOM-1 of approximately 89% was reached [43]. Seeling et al. (2006) also reported a total recovery of DON + DOM-1 of 80–89% in the effluent of a RUSITEC model [24]. The conversion of DON to its far less toxic metabolite DOM-1 at normal pH is in accordance with literature [24,28,43,44]. e.g., the Genus *novus* (formerly *Eubacterium*) species *novus* BBSH 797 of the *Coriobacteriaceae* family isolated from the rumen of bovines is able to perform this detoxification and has been developed into a commercial detoxifier, Biomin^®^ BBSH 797 [36,44,45]. EFSA approved the efficacy of this mycotoxin detoxifier in safely biotransforming trichothecenes, such as DON, into non-toxic compounds in the gastrointestinal tract of poultry and pigs, as demonstrated in numerous feeding trials [46]. As can be seen from the results in rumen fluid, this detoxification can be hampered when the pH of the rumen is low, especially with a rumen microbial community and/or activity similar to that of a non-lactating cow inoculum. In cows with SARA, periods of rumen pH depression are seen, although this is not a constant pH depression as in the performed study. A lower detoxification of DON when pH decreases has already been described by He et al. whereby the contents of the large intestine of chickens could not detoxify DON anymore in vitro when the buffer pH was lower than 5.2 [31]. In addition, Valgaeren et al. demonstrated a close relationship between the detoxification of DON and the functional ruminal microbiota [14]. By means of a toxicokinetic study the authors showed that the oral bioavailability of DON is markedly increased in non-ruminating calves (50.7%) compared to ruminating calves (4.1%). Also in our study, lower microbial activity, e.g., by inoculum of non-lactating cows and at low rumen pH, impaired DON detoxification compared to the other treatments. These findings indicate that in these conditions also in ruminants DON might stay partially intact in the rumen.

Subacute ruminal acidosis is accompanied by rumenitis which leads to a higher risk of absorption of toxic compounds, such as lipopolysaccharides, through the rumen wall [47]. Hence, in the case of SARA, it is possible that non-degraded mycotoxins are also absorbed in the rumen. In addition, the level of feed intake and feed digestibility largely influence the gastro-intestinal retention of ingesta. Hence, high yielding dairy cows that ingest high levels of highly digestible feed are more exposed to DON and other mycotoxins, as the rumen microbiota have less time to transform these toxic compounds due to a shorter retention time in the rumen. As this mycotoxin has cytotoxic effects on bovine intestinal epithelial cells (half of the maximal inhibitory concentration (IC_50_) = 1.2–3.6 µM = 0.36 µg/mL–1.1 µg/mL, depending on the performed cytotoxicity test), this mycotoxin can lead to intestinal epithelial barrier disruption [48]. Symptoms already described in ruminants related to high DON contamination of feed are gastrointestinal problems, soft stool, diarrhea, immunosuppression, decreased feed intake and a general decrease of performance [49].

We could not confirm the detoxification of NIV into its far less toxic metabolite deepoxynivalenol (de-epoxy-NIV) in the present study as the analytical standard for this metabolite is not commercially available. However, this detoxification step can be assumed as this conversion in rumen fluid has already been described in literature whereby 78–82% of incubated NIV was transformed into de-epoxy-NIV [25]. Just like DON, the detoxification can be hampered when the pH of the rumen is low (e.g., during periods of rumen pH depression in cows with SARA), especially when the rumen microbiota is less active (as indicated here by the lower VFA production by the inoculum of non-lactating cows). As such, intact NIV also potentially can reach the postruminal parts of the gastrointestinal tract. This mycotoxin shows an even higher cytotoxic effect on bovine intestinal epithelial cells than DON (IC_50_ = 0.8–1.0 µM = 0.25 µg/mL–0.31 µg/mL, depending on the performed cytotoxicity test), which may lead to similar toxic effects [48].

#### 2.3.2. Mycotoxins Partially Disappearing at Normal pH: ENN B, ROQ-C and ZEN

The final model for the ENN B concentration, expressed relative to the maximal ENN B concentration counts, just like DON and NIV, nine fixed effects model parameters with replicate as random intercept and time as random slope. An overview of the parameter estimates can be found in Table 4.

**Figure 5:**
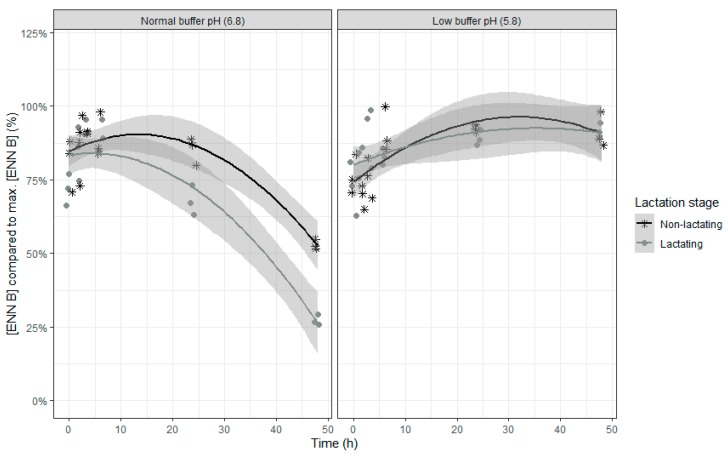
Enniatin B concentration expressed relative to the maximum ENN B concentration during an in vitro rumen simulation study. In this study, the effect of rumen fluid pH (5.8 or 6.8) and lactation stage (inoculum of non-lactating or lactating cows) on the disappearance of ENN B was investigated during an incubation period of 48 h. The grey bands correspond to the 95% confidence level interval for predictions.

An increase in the relative ENN B concentrations was seen in the beginning of the incubation, suggesting a release of adsorbed ENN B, followed by a subsequent decrease of the ENN B concentration (Table 4 and Figure 5). When the buffer with normal pH was used, the relative concentration of ENN B over time decreased more compared to the use of the low pH buffer.

These findings illustrate that the rumen microbiota at normal pH of 6.8 are able to degrade ENN B to a large extent, up to 72% after 48 h of incubation. However, when the rumen pH is low (as in SARA conditions), degradation of this mycotoxin is hampered. In addition, the type of rumen inoculum further determined the efficacy of the ENN B degradation with inoculum of non-lactating cows showing less degradation of the mycotoxin. As such, the rumen environment, both in terms of pH and microbial activity (as assessed by in vitro VFA production) can lead to a higher portion of intact ENN B reaching the postruminal parts of the gastrointestinal tract intact. This may lead to toxic effects towards intestinal cells due to the ionophoric properties of this mycotoxin [50]. Cytotoxicity of ENN B already has been demonstrated in a bovine intestinal epithelial cell line with a half maximal inhibitory concentration (IC_50_) for lysosomal activity at 4.0 µM (=2.6 µg/mL) and metabolic activity at 6.7 µM (=4.3 µg/mL) [48].

The final model for relative ROQ-C concentration compared to the maximal ROQ-C concentration counts six fixed effects model parameters and replicate as random intercept. An overview of the parameter estimates can be found in Table 5.

**Figure 6:**
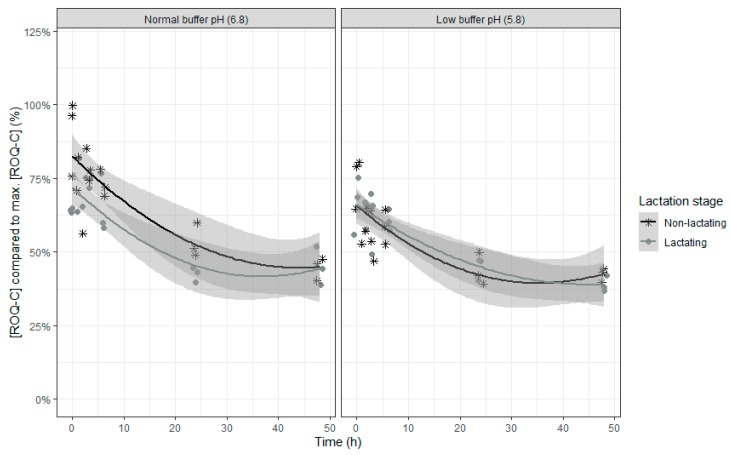
Roquefortin C concentration expressed relative to the maximum ROQ-C concentration during an in vitro rumen simulation study. In this study, the effect of rumen fluid pH (5.8 or 6.8) and lactation stage (inoculum of non-lactating or lactating cows) on the disappearance of ROQ-C was investigated during an incubation period of 48 h. The grey bands correspond to the 95% confidence level interval for predictions.

In contrast to ENN B, which also shows a partial disappearance in the rumen in vitro model, no interactions with time were seen, so the model counts three fixed effects model parameters less. The relative ROQ-C concentration decreased over time, with a slower disappearance towards the end of the incubation (Table 5 and Figure 6). When the inoculum of lactating cows was used, a higher disappearance of ROQ-C was seen. In addition, when the inoculum of lactating cows was used, a higher difference in disappearance of ROQ-C was seen between the two pH levels compared to when the inoculum of dry cows was used.

The partial degradation of ROQ-C is consistent with literature where a recovery of more than 40% of ROQ-C was reported after 48 h of incubation [30]. Although the inoculum of lactating cows shows more ROQ-C degradation, when the ruminal pH is low, the degradation capacity decreases. In literature, it has been described that ROQ-C can also be degraded by exposure to daylight [51]. When ROQ-C was dissolved in ethyl acetate and kept in diffuse daylight a half-life of 50 min was reported [51]. As the incubation flasks were not protected from daylight, the decrease in free ROQ-C concentration could be partly due to non-microbial degradation. Overall, it can be stated that the mycotoxin ROQ-C can partially reach the postruminal parts of the gastrointestinal tract. As this mycotoxin has antimicrobial properties, this can have a negative effect on the intestinal microbiota [52,53]. In addition, cytotoxic properties have also been demonstrated on intestinal epithelial cells. In Caco-2 cells, derived from a human colorectal adenocarcinoma, an IC_20_ of 100 µM (=38.9 µg/mL) was determined when exposed for 48 h to ROQ-C [54]. Although this mycotoxin also has neurotoxic properties when reaching the systemic circulation after absorption, paralytic symptoms are rarely seen [55].

The final model for relative ZEN concentration compared to the maximal ZEN concentration counts four fixed effects model parameters with replicate as random intercept. An overview of the parameter estimates can be found in Table 6.

**Figure 7:**
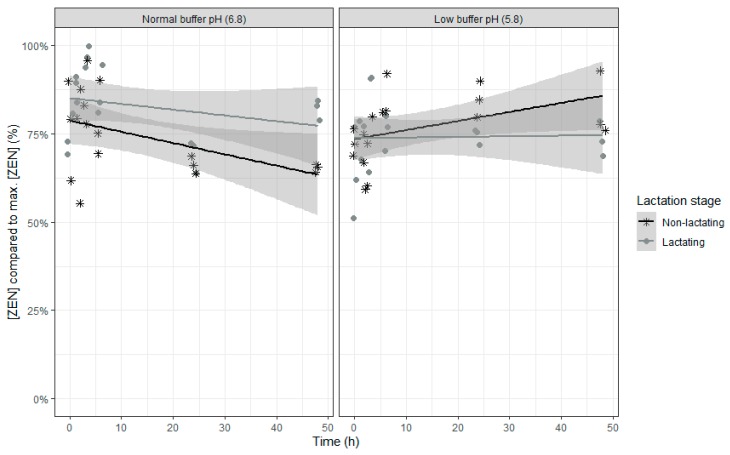
Zearalenone concentration expressed relative to the maximum ZEN concentration during an in vitro rumen simulation study. In this study, the effect of rumen fluid pH (5.8 or 6.8) and lactation stage (inoculum of non-lactating or lactating cows) on the disappearance of ZEN was investigated during an incubation period of 48 h. The grey bands correspond to the 95% confidence level interval for predictions.

For ZEN, even less fixed effects model parameters were included as time didn’t show an effect. As the trend for disappearance of ZEN over time was different at low pH compared to normal pH, the time effect disappeared. When only including the normal pH data in the model, a partial disappearance was seen over time (*p*-value time effect: 0.0101). The effect of lactation stage and the interaction of lactation stage with buffer on ZEN disappearance is opposite compared with ROQ-C. The relative ZEN concentration was higher when rumen fluid of lactating cows was used (Table 6 and Figure 7). However, in combination with a buffer with low pH, rumen fluid of lactating cows resulted in lower relative ZEN concentrations.

The final models for relative molar α-ZEL or β-ZEL concentration compared to the maximal molar ZEN concentration both count six fixed effects model parameters with replicate as random intercept and time as random slope. An overview of the parameter estimates can be found in Table 7 and Table 8.

**Figure 8:**
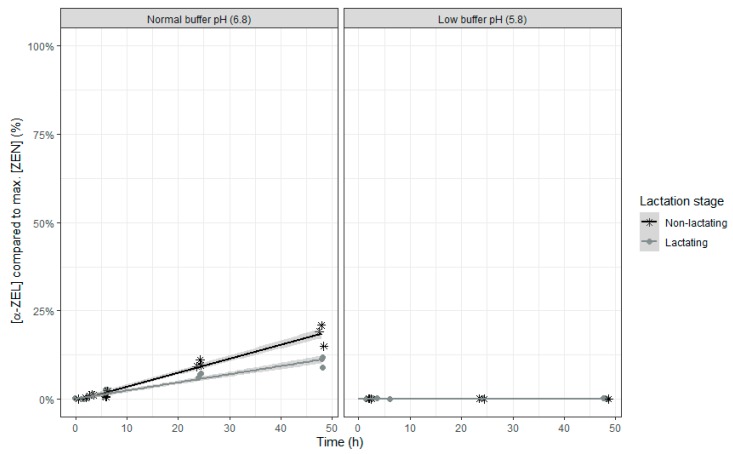
Molar α-ZEL concentration expressed relative to the maximum molar ZEN concentration during an in vitro rumen simulation study. In this study, the effect of rumen fluid pH (5.8 or 6.8) and lactation stage (inoculum of non-lactating or lactating cows) on the formation of the metabolite α-ZEL out of the parent molecule ZEN was investigated during an incubation period of 48 h. The grey bands correspond to the 95% confidence level interval for predictions.

**Figure 9:**
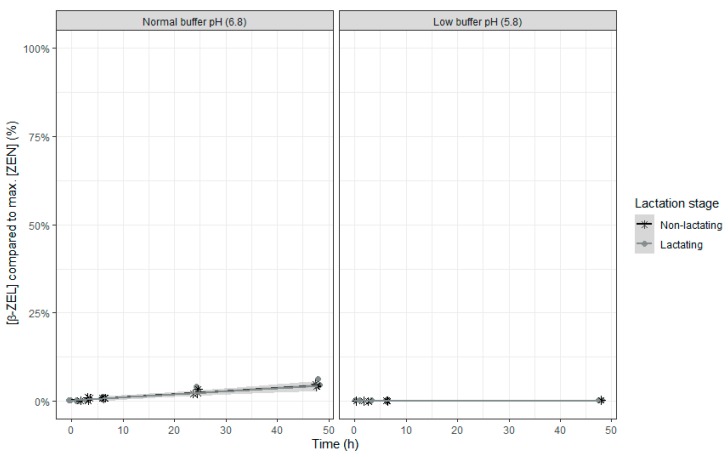
Molar β-ZEL concentration expressed relative to the maximum molar ZEN concentration during an in vitro rumen simulation study. In this study, the effect of rumen fluid pH (5.8 or 6.8) and lactation stage (inoculum of non-lactating or lactating cows) on the formation of the metabolite β-ZEL out of the parent molecule ZEN was investigated during an incubation period of 48 h. The grey bands correspond to the 95% confidence level interval for predictions.

No α-ZEL or β-ZEL metabolites were formed when the rumen fluid-buffer mixture with low pH was used (Figure 8 and Figure 9). When the rumen fluid-buffer mixture with normal pH was applied, an increased concentration of α-ZEL and β-ZEL over time was seen (Table 7 and Table 8, Figure 8 and Figure 9). A higher formation of α-ZEL over time was seen when rumen fluid of non-lactating cows was used. By contrast, no effect of inoculum was detected on formation of the metabolite β ZEL (Table 8 and Figure 9).

The metabolites ZAN, α-ZAL and β-ZAL were not detected in this study, which is in accordance with literature [28,29].

In this study, transformation of ZEN into its metabolites α-ZEL and β-ZEL occurred only when the pH of the buffer was 6.8, although this transformation was very limited, especially for β-ZEL. Transformation of ZEN to its metabolite α-ZEL is considered an activation step as this metabolite has a higher estrogenic activity compared to ZEN, whereas transformation of ZEN to its metabolite β-ZEL is considered a deactivation step as this metabolite has a lower estrogenic activity compared to ZEN [56]. In this study, a higher portion was transformed into α-ZEL compared to β-ZEL, which is also reported in literature [24,27]. Although α-ZEL is more toxic than its parent compound, its oral bioavailability is lower because of its higher polarity [57]. Results of previously performed in vitro experiments on ruminal ZEN transformation into its metabolites α-ZEL and β-ZEL are largely variable depending on the combination of the in vitro conditions and experimental setups [24,27,58,59]. It has been shown that ZEN transformation is more pronounced when the rumen inoculum was taken directly after feeding [59]. As the rumen inocula in this study were collected before morning feeding, this could be the reason why a relatively high amount of ZEN remained intact. In addition, ZEN and its metabolites α-ZEL and β-ZEL underlie a redox steady-state, which means that the detected ZEN concentration is not only the ruminally non-degraded ZEN, but also ZEN re-formed in the redox cycle [58]. In vivo studies also confirm that ZEN is partially transformed by the rumen microbiota into its metabolites α-ZEL and β-ZEL, while ZAN, α–ZAL and β-ZAL were not detectable [28,33].

As ZEN is not completely metabolized in the rumen, ZEN can enter the intestinal tract intact. Cases of hyperoestrogenism are very rare in ruminants and occur only in calves and young heifers, after ingestion of extremely high contaminated feed or after long-term exposure to ZEN [60,61,62,63,64]. As can be seen from this study, long-term exposure to ZEN is possible as ZEN is often found in maize silages and ZEN is not completely degraded in the rumen. The most characteristic symptoms of ZEN intoxication include swelling and hyperemia of the vulva, vagina and udder, enlargement of undeveloped uteri, feminization of young males and reproductive problems [49].

In conclusion, ENN B, ROQ-C, and ZEN are partially degraded in the rumen at a normal pH, but a low pH and rumen inoculum of non-lactating cows negatively affects the disappearance of ENN B and ROQ-C, while ZEN even doesn’t show any transformation at low pH.

#### 2.3.3. Mycotoxins not Disappearing at Normal pH: MPA

The final model for relative MPA concentration compared to the maximal MPA concentration counts six fixed effects model parameters and replicate as random intercept. An overview of the parameter estimates can be found in Table 9.

**Figure 10:**
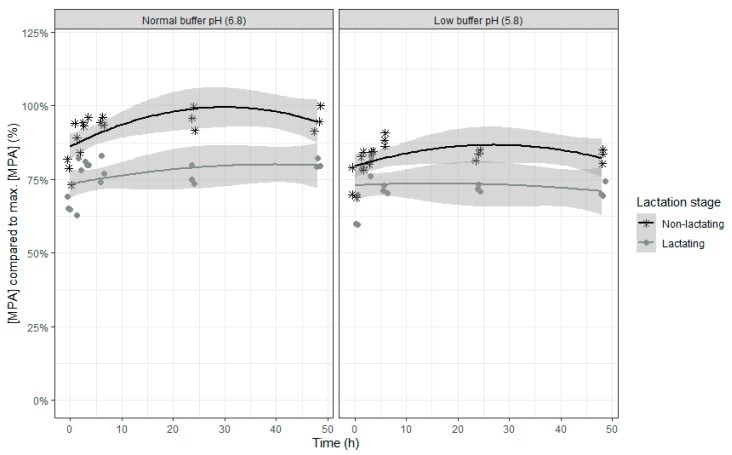
Mycophenolic acid concentration expressed relative to the maximum MPA concentration during an in vitro rumen simulation study. In this study, the effect of rumen fluid pH (5.8 or 6.8) and lactation stage (inoculum of non-lactating or lactating cows) on the disappearance of MPA was investigated during an incubation period of 48 h. The grey bands correspond to the 95% confidence level interval for predictions.

An increase in the relative MPA concentrations was seen in the beginning of the incubation, suggesting a release of bound MPA, followed by a subsequent slight decrease of the MPA concentration (Table 9 and Figure 10). As the change in MPA concentration over time is only minimal and differences between the four combinations already exist at the beginning of the incubation, it can be concluded that no biologically relevant microbial degradation occurs during the incubation. When the inoculum of lactating cows or a lower buffer pH was used, a lower concentration of MPA was seen, but the difference between inoculum of lactating and non-lactating cows was greater at normal compared with low pH (interaction effect between lactation stage and buffer).

As no relevant microbial degradation occurs during the incubation, differences in degree of adsorption is hypothesized whereby less free MPA was present when the pH was low. An overall lower MPA concentration was seen for the inoculum of lactating cows compared to the inoculum of non-lactating cows due to a higher microbial activity (demonstrated by the in vitro VFA production), which could explain the lower free MPA concentration. The partial recovery of MPA is consistent with literature where a recovery of more than 70% of MPA was reported after 48 h of incubation [30]. Although the rumen pH and inoculum may have an influence on the ruminal stability of the mycotoxin, about 75% can reach the postruminal parts of the gastrointestinal tract intact. Cytotoxic properties have already been demonstrated on intestinal epithelial cells. In Caco-2 cells, an IC_20_ of 82 µM (=26.3 µg/mL) has been determined when exposed for 48 h to MPA [54]. Besides an acute toxicity, a chronic toxicity was also seen whereby MPA induced a decrease of the barrier function after 21 days of exposure [54]. As MPA has immunosuppressive properties after absorption in the gastrointestinal tract, higher incidence of infectious diseases can be expected when ruminants ingest feed that is highly contaminated with this mycotoxin [53,65].

## 3. Conclusions

In this in vitro study, where realistic mycotoxin concentrations were used, on the one hand, no effect of mycotoxins on VFA production was seen. On the other hand, this study shows that not all mycotoxins are (fully) degraded by the rumen microbiota. ENN B and ROQ-C are only partially degraded, whereas MPA shows almost no degradation. For ZEN, a limited transformation of ZEN to α-ZEL and β-ZEL was observed at normal pH. In addition, the importance of a healthy rumen environment for the detoxification of mycotoxins is demonstrated. In case of rumen acidosis and/or a lower activity of the rumen microbiota as occurring in non-lactating cows, the degradation of ENN B, DON and NIV is hampered. This emphasizes the importance of mycotoxin control in ruminants, especially in high yielding dairy cows that often suffer from SARA. In conclusion, also in ruminants, mycotoxins can enter the postruminal parts of the gastrointestinal tract intact and exert their negative effects in the animals.

## 4. Materials and Methods

### 4.1. Rumen Fluid, Maize Silage, Mycotoxins, Chemicals and Reagents

Rumen fluid was collected from four fistulated Holstein ruminants (Institute for Agricultural and Fisheries and Food Research, EC2014/241) prior to the morning feeding. The cows were between 6–7 years old. Cow 1 and cow 2 were 250 and 110 days in lactation and had a milk production of 36.5 and 50.5 L of milk per day, respectively. Both cows got 50% of maize silage and 50% of wilted grass silage on dry matter basis ad libitum per day, supplemented with concentrate according to milk production. Cow 3 and 4 were not lactating and got 8–9 kg of maize silage on dry matter basis and 4.1 kg of chopped straw per day, supplemented with 1 kg of concentrate. The collected rumen fluid was immediately transferred from the barn to the lab in thermos flasks and the pH was measured before making the buffer-rumen fluid mixture (see Section 4.2). The rumen fluid had a pH of 6.16, 5.38, 6.64 and 6.71 for cow 1, 2, 3 and 4, respectively.

Maize silage, as substrate for the incubation, was obtained from Agrivet (Melle, Belgium) in May 2012, lyophilized and stored at ambient temperature as standard incubation substrate. Mycotoxin concentrations were determined in the lyophilized maize silage sample at the Centre of Excellence in Mycotoxicology and Public Health, Department of Bioanalysis at Ghent University (Belgium) using LC-MS/MS. Only traces of ROQ-C (12 µg/kg), ZEN (68 µg/kg), NIV (201 µg/kg), and DON (593 µg/kg) were detected. The concentration of ENN B was below the cut-off value of 80 µg/kg.

The analytical standards of DON, NIV, MPA, ROQ-C and ZEN were purchased from Fermentek (Jerusalem, Israel). The standards of DOM-1, ENN B, ZAN, α-ZEL, β-ZEL, α-ZAL and β-ZAL were purchased from Sigma-Aldrich (Overijse, Belgium). The internal standards (IS) ^13^C_17_-MPA, ^13^C_22_-ROQ-C and ^13^C_18_-ZEN were purchased from Food Risk Management (Oostvoorne, The Netherlands). The IS ^13^C_15_-DON was purchased from Sigma-Aldrich. The IS ^15^N_3_-ENN B was kindly donated by prof. dr. Michael Rychlik (Technical University of Munich, Freising, Germany) [66].

Methanol (MeOH), acetonitrile (ACN) and water (H_2_O) were purchased from Biosolve (Valkenswaard, The Netherlands) and were of ULC/MS grade. Acetic acid (AA), hydrochloric acid (HCl) 37%, ethanol and ethyl acetate (EtAc) were purchased from Merck Millipore (Overijse, Belgium) and were of analytical grade. Ammonium hydrogen carbonate (NH_4_HCO_3_) was purchased from VWR (Leuven, Belgium). The phosphate-buffered saline (PBS) powder packs were purchased from Thermo Fisher Scientific (Merelbeke, Belgium). Magnesium chloride hexahydrate (MgCl_2_.6H_2_O) was purchased from Carl Roth (Vienna, Austria). MES (2-(N-morpholino)ethanesulfonic acid, 4-morpholineethanesulfonic acid), sodium hydrogen carbonate (NaHCO_3_) and 2-ethylbutyric acid (EBA) were purchased from Sigma-Aldrich (Overijse, Belgium). Formic acid was purchased from UCB (RPL, Leuven, Belgium). Carbon dioxide (CO_2_), was purchased from Air Liquide (Aalter, Belgium).

### 4.2. Preparation of Standard Solutions and Buffer-Rumen Fluid Mixture

Stock solutions of 1 mg/mL in ACN were prepared for DON, NIV, MPA, ROQ-C, ZEN, and ENN B and of 100 μg/mL in ACN for ZAN, α-ZEL, β-ZEL, α-ZAL and β-ZAL. DOM-1 was already in solution upon purchase (50 μg/mL ACN). An IS-stock solution of 5 μg/mL in ACN was prepared for ^15^N_3_-ENN B. All other IS were already in solution upon purchase (^13^C_15_-DON: 25.3 μg/mL in ACN, ^13^C_17_-MPA: 25.4 μg/mL in ACN, ^13^C_22_-ROQ-C: 25 μg/mL in ACN, ^13^C_18_-ZEN: 25.4 μg/mL in ACN). The standard and IS stock solutions were used to make working solutions for the preparation of spiking solutions (see Section 4.3), matrix-matched calibrator and quality control (QC) samples (see Section 4.5). All stock and working solutions were stored at ≤−15 °C.

To determine VFA concentrations, an IS solution was made containing 10 mg of EBA in 10 mL formic acid.

The buffer for the in vitro rumen simulation contained the following compounds per liter: 54.31 g MES, 0.124 g MgCl_2_.6H_2_O, 8.74 g NaHCO_3_, and 1.00 g NH_4_HCO_3_. The buffer was saturated with CO_2_ overnight and kept at 39 °C. Before adding rumen fluid, pH was adjusted with 6M of HCl to 6.8 or 5.8. Fresh rumen fluid of two lactating cows was sieved using a sieve with a mesh width of 1 mm, mixed together and added to the buffer with low pH (L5.8, final pH = 5.87) and the buffer with normal pH (L6.8, final pH = 6.76). In addition, fresh rumen fluid of two non-lactating cows was sieved, mixed together and added to the buffer with low pH (D5.8, final pH = 5.93) and the buffer with normal pH (D6.8, final pH = 6.87). The rumen fluid/buffer ratio of all four combinations was 263.2 mL/1000 mL.

### 4.3. In vitro Rumen Simulation Experiment

A spiking solution was made and contained 150 µg/mL NIV, 30 µg/mL DON, 2.5 µg/mL ENN B, 15 µg/mL MPA, 5 µg/mL ROQ-C and 7.5 µg/mL ZEN, dissolved in an ethanol/H_2_O mixture (50/50, *v*/*v*). Twenty µL of the spiking solution was added to 50 mg of maize silage in an incubation flask of 25 mL, so the maize silage contained 60 mg/kg NIV, 12 mg/kg DON, 1 mg/kg ENN B, 6 mg/kg MPA, 2 mg/kg ROQ-C and 3 mg/kg ZEN. For DON and ZEN, the concentrations were based on the maximum guidance values in maize by-products formulated by the Commission of the European Communities [20]. As no maximum guidance values are available for the other mycotoxins, the concentrations of those mycotoxins were based on worst case contamination levels in maize silage found in Belgium and the Netherlands described in literature [12,19,21,22,23]. After adding 200 µL of distilled water, the incubation flasks were flushed with CO_2_ and 4.8 mL of fresh buffer-rumen fluid mixture was added. The samples were incubated in triplicate at 39 °C in a shaking incubator (Edmund Bühler TH30; Edmund Bühler GmbH, Hechingen, Germany). After 1.5 h, 3 h, 6 h, 24 h, and 48 h, two mL of the rumen fluid sample was taken to determine VFA concentration and 250 µL to determine mycotoxin concentrations. In addition, pH was measured from each sample to confirm pH stability throughout the incubation.

### 4.4. Determination of VFA Concentration

Total VFA production was calculated as the sum of acetic acid, propionic acid, isobutyric acid, butyric acid, isovaleric acid, valeric acid and caproic acid. To determine these VFA concentrations, 200 µL of the EBA IS solution was added to 2.0 mL of rumen fluid sample. The sample was centrifuged at 30,996× *g* at 4 °C for 15 min. The supernatant was filtered over glass wool and transferred into an autosampler vial. Samples were stored at 4 °C until analysis by means of gas chromatography (HP 7890A, Agilent Technologies, Diegem, Belgium) equipped with a flame ionization detector and a Supelco Nukol capillary column (30 m × 0.25 mm × 0.25 μm, Sigma-Aldrich, Diegem, Belgium), as described in Jeyanathan et al. [67].

### 4.5. Preparation of Calibrator and Quality Control (QC) Samples Used for Mycotoxin Analysis

Matrix-matched calibration curves were prepared for each combination of rumen fluid-buffer mixture (L5.8, L6.8, D5.8, D6.8). The appropriate combined working solution was added to 250 µL of rumen fluid-buffer mixture to obtain a calibration range of 0.6–180 ng/mL of DON and DOM-1, 30–900 ng/mL of NIV, 0.05–15 ng/mL of ENN B, 0.3–90 ng/mL of MPA, 0.10–30 ng/mL of ROQ-C and 0.15–45 ng/mL of ZEN, ZAN, α-ZEL, β-ZEL, α-ZAL and β-ZAL. Quality control samples were prepared at medium (10% of the start concentration in spiked samples) and high (start concentration in spiked samples) concentration levels.

### 4.6. Rumen Fluid Sample Extraction and Mycotoxin Analysis

The procedure described in Debevere et al. [68] was applied to extract DON, DOM-1, NIV, ENN B, MPA, ROQ-C, ZEN, and possible metabolites from the in vitro rumen study samples and from the matrix-matched calibrator and QC samples. In brief, 25 µL of the IS-mix working solution (100 ng/mL for ^13^C_15_-DON, ^13^C_17_-MPA, and ^13^C_18_-ZEN, and 10 ng/mL for ^15^N_3_-ENN B and ^13^C_22_-ROQ-C), 250 µL of PBS and 1.5 mL of EtAc were added to the 250-µL rumen fluid sample. The samples were vortex mixed and extracted on an overhead shaker (Trayster digital, IKA, Staufen, Germany) for 15 min. After centrifugation (3724× *g*, 5 min, 4 °C), the upper organic phase was collected and evaporated to dryness under a gentle nitrogen stream at ~50 °C. The dry residue was redissolved in 200 µL of H_2_O/ACN (85/15, *v*/*v*), vortex mixed, filtrated using a 0.20 µm Millex^®^-LG PTFE filter (Merck Millipore, Overijse, Belgium) and collected in an autosampler vial.

The ultra-high performance liquid chromatography (UPLC) system consisted of an Acquity H-Class Quaternary Solvent Manager and Flow-Through-Needle Sample Manager with temperature controlled tray and column oven from Waters (Zellik, Belgium). Chromatographic separation of the analytes was achieved on an Acquity UPLC^®^ HSS T3 column (100 mm × 2.1 mm I.D., particle size (dp): 1.8 µm) in combination with an Acquity HSS T3 Vanguard pre-column (5 mm × 2.1 mm I.D., dp: 1.8 µm) both from Waters. The UPLC system was coupled to a Xevo^®^ TQ-S MS/MS system, equipped with an ESI probe operating in both the positive and negative ionization mode (all from Waters). Liquid chromatography and mass spectrometric parameters were optimized as described in Debevere et al. [68]. The developed UPLC-MS/MS method was validated in-house by a set of parameters that were in compliance with the recommendations as defined by the European Community [69] and with reference guidelines defined in other EU and FDA documents [70,71,72]. The method validation protocol and the acceptance criteria are reported in detail in Debevere et al. [68].

MassLynx version 4.1 software was used for data processing (Copyright^©^ 2012, Waters, Zellik, Belgium).

### 4.7. Data Modeling and Statistical Analysis

Experiments to determine the effect of pH and lactation stage on the disappearance of mycotoxins in the rumen as well as the effect of pH, lactation stage, and mycotoxins on VFA production were performed in triplicate and all incubations were done in the same run. Statistical analyses were performed with RStudio version 1.1.442 (Copyright^©^ 2009-2018 R studio Inc., Boston, MA, USA) using R version 3.4.4. For analysis of mycotoxin disappearance, multilevel analysis was performed using step forward model building including random intercept of replicate, time, and time² as first level, pH, lactation stage of inoculum donors and their interaction as second level, random slope of time and two-way interactions between time and second level effects. All lower level interactions were included in the model if a higher level interaction was significant. Random factors (replicates and time) had no/limited effect on the model. For analyses of VFA production, the presence of mycotoxins was introduced into the model as a second level effect. All analyses were performed using the lmer function in the lme4 package version 1.1–17 [73]. The Akaike information criterion (AIC) was used to judge the model fit. For each model, R^2^_m_ (marginal R^2^: variance explained by fixed factors) was calculated using the MuMIn-package version 1.43.6 [74]. In the tables, model estimators are represented with standard error (SE). Values of *p* < 0.05 were considered statistically significant. Graphs were constructed using ggplot2 package version 2.2.1 [75].

## Figures and Tables

**Table 1:** Overview of parameter estimates of the model to determine total net volatile fatty acid (VFA) production (µmol) during an in vitro rumen simulation study. In this study, the effect of rumen fluid-buffer pH (5.8 or 6.8), lactation stage (inoculum of non-lactating or lactating cows) and the presence or absence of mycotoxins as well as the incubation time on the total VFA production was investigated during an incubation period of 48 h. R^2^_m_ = 0.985. Time^2^ means time squared.

Fixed Effect	Estimate	SE	*p*-Value
Intercept	−24.4	2.79	<0.001
Time	12.1	0.28	<0.001
Time^2^	−0.140	5.6 × 10^−3^	<0.001
Lactation stage (lactating)	24.7	2.27	<0.001
Buffer (pH 5.8)	−14.2	2.27	<0.001
Mycotoxins (present)	−0.0320	2.27030	0.989

**Table 2:** Overview of parameter estimates of the model to determine the DON and NIV concentration expressed relative to the maximal DON or NIV concentration during an in vitro rumen simulation study. In this study, the effect of rumen fluid-buffer pH (5.8 or 6.8) and lactation stage (inoculum of non-lactating or lactating cows) on disappearance of DON and NIV was investigated during an incubation period of 48 h. R^2^_m_ = 0.933 for DON and 0.912 for NIV. Time^2^ means time squared.

Figure	DON			NIV		
	Estimate	SE	*p*-Value	Estimate	SE	*p*-Value
Intercept	8.82 × 10^−1^	2.89 × 10^−2^	<0.001	8.48 × 10^−1^	3.08 × 10^−2^	<0.001
Time	−4.24 × 10^−2^	2.834 × 10^−3^	<0.001	−3.44 × 10^−2^	3.08 × 10^−3^	<0.001
Time^2^	4.72 × 10^−4^	5.40 × 10^−5^	<0.001	3.12 × 10^−4^	5.75 × 10^−5^	<0.001
Lactation stage (lactating)	−1.08 × 10^−1^	3.90 × 10^−2^	0.00841	−1.12 × 10^−1^	4.15 × 10^−2^	0.00716
Buffer (pH 5.8)	1.08 × 10^−1^	3.90 × 10^−2^	0.00566	2.60 × 10^−2^	4.152 × 10^−2^	0.531
Lactation stage × Buffer (lactating × pH 5.8)	3.38 × 10^−2^	5.513 × 10^−2^	0.540	1.29 × 10^−1^	5.87 × 10^−2^	0.0284
Time × Lactation stage (Time × lactating)	2.46 × 10^−3^	1.750 × 10^−3^	0.163	2.59 × 10^−3^	1.880 × 10^−3^	0.169
Time × Buffer (Time × pH 5.8)	1.39 × 10^−2^	1.77 × 10^−3^	<0.001	1.75 × 10^−2^	1.88 × 10^−3^	<0.001
Time × Lactation stage × Buffer (Time × lactating × pH 5.8)	−1.62 × 10^−2^	2.50 × 10^−3^	<0.001	−1.86 × 10^−2^	2.66 × 10^−3^	<0.001

**Table 3:** Overview of parameter estimates of the model to determine the DOM-1 molar concentration expressed relative to the maximal DON molar concentration during an in vitro rumen simulation study. In this study, the effect of rumen fluid-buffer pH (5.8 or 6.8) and lactation stage (inoculum of non-lactating or lactating cows) on formation of the metabolite DOM-1 out of the parent molecule DON was investigated during an incubation period of 48 h. R^2^_m_ = 0.951. Time^2^ means time squared.

Fixed Effect	Estimate	SE	*p*-Value
Intercept	−1.40 × 10^−2^	2.100 × 10^−2^	0.494
Time	3.53 × 10^−2^	2.06 × 10^−3^	<0.001
Time^2^	−3.61 × 10^−4^	3.90 × 10^−5^	<0.001
Lactation stage (lactating)	−1.12 × 10^−3^	2.8310 × 10^−2^	0.969
Buffer (pH 5.8)	−5.03 × 10^−2^	2.831 × 10^−2^	0.0759
Lactation stage × Buffer (lactating × pH 5.8)	2.98 × 10^−3^	2.0040 × 10^−2^	0.941
Time × Lactation stage (Time × lactating)	−1.70 × 10^−3^	1.280 × 10^−3^	0.196
Time × Buffer (Time × pH 5.8)	−1.21 × 10^−2^	1.28 × 10^−3^	<0.001
Time × Lactation stage × Buffer (Time × lactating × pH 5.8)	1.30 × 10^−2^	1.81 × 10^−3^	<0.001

**Table 4:** Overview of parameter estimates of the model to determine the ENN B concentration expressed relative to the maximal ENN B concentration during an in vitro rumen simulation study. In this study, the effect of rumen fluid-buffer pH (5.8 or 6.8) and lactation stage (inoculum of non-lactating or lactating cows) on disappearance of ENN B was investigated during an incubation period of 48 h. R^2^_m_ = 0.782. Time^2^ means time squared.

Fixed Effect	Estimate	SE	*p*-Value
Intercept	0.860	0.0231	<0.001
Time	4.59 × 10^−3^	2.269 × 10^−3^	0.0429
Time^2^	−2.33 × 10^−4^	4.327 × 10^−4^	<0.001
Lactation stage (lactating)	−0.0154	0.03122	0.620
Buffer (pH 5.8)	−0.120	0.0313	<0.001
Lactation stage × Buffer (lactating × pH 5.8)	0.0550	0.00415	0.213
Time × Lactation stage (Time × lactating)	−5.22 × 10^−3^	1.433 × 10^−3^	<0.001
Time × Buffer (Time × pH 5.8)	0.0100	0.00141	<0.001
Time × Lactation stage × Buffer (Time × lactating × pH 5.8)	3.92 × 10^−3^	2.000 × 10^−3^	0.0499

**Table 5:** Overview of parameter estimates of the model to determine the ROQ-C concentration expressed relative to the maximal ROQ-C concentration during an in vitro rumen simulation study. In this study, the effect of rumen fluid-buffer pH (5.8 or 6.8) and lactation stage (inoculum of non-lactating or lactating cows) on disappearance of ROQ-C was investigated during an incubation period of 48 h. R^2^_m_ = 0.732. Time^2^ means time squared.

Fixed Effect	Estimate	SE	*p*-Value
Intercept	0.799	0.02511	<0.001
Time	−0.0151	0.00217	<0.001
Time^2^	1.90 × 10^−4^	4.48 × 10^−5^	<0.001
Lactation stage (lactating)	−0.0822	0.02527	0.00114
Buffer (pH 5.8)	−0.129	0.0253	<0.001
Lactation stage × Buffer (lactating × pH 5.8)	0.0883	0.03574	0.0134

**Table 6:** Overview of parameter estimates of the model to determine the ZEN concentration expressed relative to the maximal ZEN concentration during an in vitro rumen simulation study. In this study, the effect of rumen fluid-buffer pH (5.8 or 6.8) and lactation stage (inoculum of non-lactating or lactating cows) on ZEN disappearance was investigated during an incubation period of 48 h. R^2^_m_ = 0.113.

Fixed Effect	Estimate	SE	*p*-Value
Intercept	7.44 × 10^−1^	2.35 × 10^−2^	<0.001
Lactation stage (lactating)	8.40 × 10^−2^	3.300 × 10^−2^	0.0112
Buffer (pH 5.8)	2.60 × 10^−2^	3.300 × 10^−2^	0.433
Lactation stage × Buffer (lactating × pH 5.8)	−1.15 × 10^−1^	1.70 × 10^−2^	0.0146

**Table 7:** Overview of parameter estimates of the model to determine the molar α-ZEL concentration expressed relative to the maximal molar ZEN concentration during an in vitro rumen simulation study. In this study, the effect of rumen fluid-buffer pH (5.8 or 6.8) and lactation stage (inoculum of non-lactating or lactating cows) on formation of the metabolite α-ZEL out of the parent molecule ZEN was investigated during an incubation period of 48 h. R^2^_m_ = 0.939.

Fixed Effect	Estimate	SE	*p*-Value
Intercept	−2.24 × 10^−3^	1.480 × 10^−3^	0.13
Time	3.55 × 10^−3^	2.34 × 10^−4^	<0.001
Lactation stage (lactating)	2.10 × 10^−3^	1.710 × 10^−3^	0.220
Buffer (pH 5.8)	1.19 × 10^−3^	1.710 × 10^−3^	0.486
Time × Lactation stage (lactating)	−8.19 × 10^−3^	2.70 × 10^−4^	0.00241
Time × Buffer (pH 5.8)	−3.14 × 10^−3^	2.70 × 10^−4^	<0.001

**Table 8:** Overview of parameter estimates of the model to determine the molar β-ZEL concentration expressed relative to the maximal molar ZEN concentration during an in vitro rumen simulation study. In this study, the effect of rumen fluid-buffer pH (5.8 or 6.8) and lactation stage (inoculum of non-lactating or lactating cows) on formation of the metabolite β-ZEL out of the parent molecule ZEN was investigated during an incubation period of 48 h. R^2^_m_ = 0.792.

Fixed Effect	Estimate	SE	*p*-Value
Intercept	1.78 × 10^−3^	1.100 × 10^−3^	0.106
Time	8.71 × 10^−4^	1.220 × 10^−4^	<0.001
Lactation stage (lactating)	−7.00 × 10^−5^	1.27000 × 10^−3^	0.956
Buffer (pH 5.8)	−1.75 × 10^−3^	1.270 × 10^−3^	0.169
Time × Lactation stage (lactating)	−1.50 × 10^−5^	1.4000 × 10^−4^	0.914
Time × Buffer (pH 5.8)	−8.63 × 10^−4^	1.400 × 10^−4^	<0.001

**Table 9:** Overview of parameter estimates of the model to determine the MPA concentration expressed relative to the maximal MPA concentration during an in vitro rumen simulation study. In this study, the effect of rumen fluid-buffer pH (5.8 or 6.8) and lactation stage (inoculum of non-lactating or lactating cows) on disappearance of MPA was investigated during an incubation period of 48 h. R^2^_m_ = 0.617. Time^2^ means time squared.

Fixed Effect	Estimate	SE	*p*-Value
Intercept	0.887	0.015714	<0.001
Time	4.58 × 10^−3^	1.662 × 10^−3^	0.00582
Time^2^	−7.86 × 10^−5^	3.429 × 10^−5^	0.0219
Lactation stage (lactating)	−0.152	0.0194	<0.001
Buffer (pH 5.8)	−0.0917	0.01936	<0.001
Lactation stage × Buffer (lactating × pH 5.8)	0.0606	0.03738	0.0270

## Supplementary Materials

The following are available online at https://www.mdpi.com/2072-6651/12/2/101/s1, Table S1: Mycotoxin concentrations during an in vitro rumen study. In this study, the effect of buffer pH (5.8 or 6.8) and lactation stage (inoculum of non-lactating (NL) or lactating (L) cows) on the disappearance of mycotoxins was investigated during an incubation period of 48 h. This study was performed in triplicate. The spiking levels were 120 ng/mL for DON, 600 ng/mL for NIV, 10 ng/mL for ENN B, 20 ng/mL for ROQ-C, 30 ng/mL for ZEN and 60 ng/mL for MPA.DON = deoxynivalenol, DOM-1 = deepoxy DON, NIV = nivalenol, ENN B = enniatin B, ROQ-C = roquefortine C, ZEN = zearalenone, α-ZEL = α-zearalenol, MPA = mycophenolic acid, LOD = limit.

## Abbreviations

α-ZAL	α-zearalanol
α-ZEL	α-zearalenol
β-ZAL	β-zearalanol
β-ZEL	β-zearalenol
DM	dry matter
DOM-1	deepoxy-DON
DON	deoxynivalenol
ENN B	enniatin B
MPA	mycophenolic acid
NIV	nivalenol
ROQ-C	roquefortine C
SARA	subacute ruminal acidosis
VFA	volatile fatty acid
ZAN	zearalenone
ZEN	zearalenone
