# Supplementary material for: In Vitro Rumen Simulations Show a Reduced Disappearance of Deoxynivalenol, Nivalenol and Enniatin B at Conditions of Rumen Acidosis and Lower Microbial Activity
Source: Toxins (Basel). 2020 Feb 5;12(2):101. doi: 10.3390/toxins12020101 (PMC7076776; doi:10.3390/toxins12020101)
Supplement: Supplementary file 1 [file toxins-12-00101-s001.pdf]

Supplementary Materials: In Vitro Rumen Simulations Show a Reduced Disappearance of Deoxynivalenol, Nivalenol and Enniatin B at Conditions of Rumen Acidosis and Lower Microbial Activity

Sandra Debevere, An Cools, Siegrid De Baere, Geert Haesaert, Michael Rychlik, Siska Croubels and Veerle Fievez *

Table S1. Mycotoxin concentrations during an in vitro rumen study. In this study, the effect of buffer pH (5.8 or 6.8) and lactation stage (inoculum of non-lactating (NL) or lactating (L) cows) on the disappearance of mycotoxins was investigated during an incubation period of 48 h. This study was performed in triplicate. The spiking levels were 120 ng/mL for DON, 600 ng/mL for NIV, 10 ng/mL for ENN B, 20 ng/mL for ROQ-C, 30 ng/mL for ZEN and 60 ng/mL for MPA.DON = deoxynivalenol, DOM-1 = deepoxy DON, NIV = nivalenol, ENN B = enniatin B, ROQ-C = roquefortine C, ZEN = zearalenone, α-ZEL = α-zearalenol, MPA = mycophenolic acid, LOD = limit.

Incubation time	Inoculum	Buffer pH	Replicate	DON (ng/mL)	DOM-1 (ng/mL)	NIV (ng/mL)	ENN B (ng/mL)	ROQ-C (ng/mL)	ZEN (ng/mL)	α-ZEL (ng/mL)	MPA (ng/mL)
0 h	L	6.8	1	106	<LOD	479	7.66	13.1	24.4	<LOD	41.2
			2	98.0	<LOD	422	6.64	12.7	20.7	<LOD	38.8
			3	99.4	<LOD	492	7.19	12.8	21.9	<LOD	39.0
	L	5.8	1	107	<LOD	512	8.13	14.9	23.0	<LOD	41.7
			2	106	<LOD	498	6.25	11.1	15.3	<LOD	35.8
			3	105	<LOD	567	7.27	13.7	18.6	<LOD	35.9
	NL	6.8	1	107	<LOD	481	7.11	15.2	18.5	<LOD	43.9
			2	109	<LOD	486	8.44	19.2	23.8	<LOD	48.9
			3	106	<LOD	511	8.75	20.0	26.9	<LOD	47.6
	NL	5.8	1	105	<LOD	470	7.11	13.0	20.8	<LOD	41.4
			2	104	<LOD	438	7.50	16.0	21.5	<LOD	47.1
			3	107	<LOD	492	8.36	15.7	22.9	<LOD	42.2
1.5 h	L	6.8	1	97.0	0.973	455	9.30	16.3	27.2	<LOD	49.1
			2	91.2	0.892	420	8.59	13.1	26.8	<LOD	46.8
			3	67.9	0.487	357	7.50	12.8	25.2	<LOD	37.8
	L	5.8	1	107	0.405	482	8.59	13.2	23.1	<LOD	46.6
			2	105	0.405	535	8.36	13.4	23.7	<LOD	47.2

			3	103	0.487	472	7.58	11.6	20.5	<LOD	48.0
	NL	6.8	1	97.4	0.487	475	8.75	14.3	23.8	<LOD	56.2
			2	108	0.405	535	7.27	11.1	16.6	<LOD	50.6
			3	102	0.405	477	9.14	16.3	26.3	<LOD	53.3
	NL	5.8	1	103	0.649	476	7.34	13.1	22.5	<LOD	49.7
			2	114	0.649	492	7.03	11.3	20.1	<LOD	50.6
			3	101	0.730	453	6.48	10.6	17.6	<LOD	46.8
3 h	L	6.8	1	82.6	4.70	424	9.14	15.1	28.3	<LOD	48.5
			2	87.4	5.43	416	9.53	14.3	29.1	<LOD	48.0
			3	86.1	3.73	454	9.06	15.1	30.0	<LOD	48.3
	L	5.8	1	99.8	1.38	503	7.89	9.83	19.2	<LOD	45.7
			2	101	1.30	504	9.61	13.2	27.2	<LOD	48.7
			3	97.9	1.62	467	9.92	13.9	27.3	<LOD	49.7
	NL	6.8	1	93.4	3.65	434	9.06	15.5	24.9	0.345	55.6
			2	93.6	3.08	476	9.14	14.8	23.5	0.345	56.1
			3	101	3.32	494	9.69	17.0	28.8	0.345	57.8
	NL	5.8	1	104	1.30	482	7.58	10.8	21.7	<LOD	50.3
			2	120	1.95	499	8.20	12.7	23.9	<LOD	50.8
			3	107	1.38	482	6.88	9.34	18.1	<LOD	48.0
6 h	L	6.8	1	63.2	19.2	319	8.52	11.7	24.2	0.431	46.4
			2	53.1	19.1	292	8.91	11.9	25.2	0.776	44.5
			3	60.3	23.9	328	9.53	15.4	28.1	0.776	50.1
	L	5.8	1	85.6	9.00	397	7.97	12.0	21.1	<LOD	42.7
			2	80.9	7.87	415	8.59	12.9	24.0	<LOD	41.8
			3	90.4	7.22	408	8.28	11.4	23.2	<LOD	44.1
	NL	6.8	1	76.6	19.3	469	9.84	15.6	27.0	0.604	57.9
			2	81.5	13.9	469	8.44	13.8	20.7	0.431	55.8
			3	66.2	26.1	382	8.59	14.4	22.4	0.259	56.3
	NL	5.8	1	105	3.00	526	8.83	11.8	24.4	<LOD	52.9
			2	104	2.51	488	8.52	10.5	24.4	<LOD	54.7
			3	105	3.00	501	10.0	12.7	27.7	<LOD	51.3

			1	<LOD	72.7	<LOD	7.34	9.00	21.6	2.16	47.8
24 h	L	6.8	2	<LOD	70.2	<LOD	6.33	8.51	19.2	2.16	44.6
			3	<LOD	68.3	<LOD	6.72	8.10	21.5	1.81	45.2
			1	27.6	53.8	258	8.67	7.96	22.4	<LOD	43.0
	L	5.8	2	7.0	65.0	164	8.91	9.41	21.7	<LOD	43.7
			3	11.1	64.0	184	9.22	9.48	22.9	<LOD	42.5
			1	<LOD	88.8	<LOD	8.67	9.90	19.8	2.85	59.8
	NL	6.8	2	<LOD	82.4	<LOD	8.05	10.2	19.2	2.93	55.4
			3	<LOD	83.2	<LOD	8.91	12.0	20.8	3.28	57.4
			1	108	6.65	522	9.38	7.75	26.9	<LOD	50.9
	NL	5.8	2	103	16.7	600	9.22	8.37	23.9	<LOD	50.3
			3	91.8	14.8	469	9.06	10.0	25.5	<LOD	48.7
			1	<LOD	79.4	<LOD	2.58	10.3	23.6	3.71	49.3
48 h	L	6.8	2	<LOD	76.9	<LOD	2.66	8.86	25.3	3.28	47.7
			3	<LOD	81.9	<LOD	2.89	7.89	24.9	2.76	48.2
			1	<LOD	84.2	<LOD	9.38	8.37	23.7	<LOD	41.8
	L	5.8	2	<LOD	81.9	<LOD	9.14	7.68	22.0	<LOD	42.3
			3	<LOD	85.0	<LOD	8.91	7.47	20.7	<LOD	44.5
			1	<LOD	79.7	<LOD	5.47	7.96	19.8	4.66	57.1
	NL	6.8	2	<LOD	83.4	<LOD	5.23	9.13	19.2	5.78	54.5
			3	<LOD	88.1	<LOD	5.23	9.69	19.7	6.38	60.0
			1	62.3	37.6	394	8.67	8.58	23.5	<LOD	47.9
	NL	5.8	2	61.4	35.1	388	8.91	7.96	22.9	<LOD	51.0
			3	82.6	26.0	388	9.77	8.79	28.0	<LOD	50.6
